# Supplementary material for: Changes in the Transmission Dynamic of Chikungunya Virus in Southeastern Senegal
Source: Viruses. 2020 Feb 10;12(2):196. doi: 10.3390/v12020196 (PMC7077306; doi:10.3390/v12020196)
Supplement: Supplementary file 1 [file viruses-12-00196-s001.pdf]

Supplementary Materials:

Environmental data

A set of environmental, topographical and demographic datasets was used to explore potential drivers of CHIKV outbreaks in the study area. Due to the relatively small study area, we resorted to high resolution satellite images provided by the Moderate Resolution Imaging Spectroradiometer (MODIS) instrument operating on the Terra spacecraft (NASA) [33], which measures 36 spectral bands and it acquires data at its lowest spatial resolution of 250m. From the family of MODIS products, we downloaded global MOD13Q1 data, which are provided every 16 days at 250m spatial resolution. The MOD13Q1 product includes vegetation indices such as Normalized Difference Vegetation Index (NDVI) and Enhanced Vegetation Index (EVI). The latter minimizes canopy background variations and maintains sensitivity over dense vegetation conditions. It also includes mid-infrared band (MIR) which has been found to be useful to discriminate water surfaces; water highly reflects wavelengths in the range of the MIR band (2.1 μm) [34]. Fortnightly, continuous gridded maps of NDVI, EVI and MIR for the study area were produced for 2009 and aggregated by calculating the mean, maximum and standard deviation of the rainy season (May to December).

Forest cover for the study area was obtained from the Global Forest Change project (University of Maryland) [36]. This project, which was conceived to monitor global forest extent, provides gridded maps of forest and non-forest areas based on high-resolution satellite images obtained by Landsat mission between 2000 and 2014. We later calculated the Euclidean distance (straight line distance) in kilometers from the communities to the nearest forest area. Likewise, we produced a continuous surface of distances in km to the nearest water body based on the Global Database of Lakes, Reservoirs and Wetlands.

The elevation dataset at 250m resolution was derived from a gridded digital elevation model produced by the Shuttle Radar Topography Mission (SRTM) [39]. This elevation surface was then processed to obtain slope in degrees. In addition, a gridded map of urban accessibility at 1 km resolution was obtained from the European Commission Joint Research Centre Global Environment Monitoring Unit (JRC) [44]. This dataset defined urban accessibility as the predicted time taken to travel from that grid cell to a city of ≥50,000 persons in the year 2000 using land- or water-based travel. Finally, gridded maps at 100m resolution of estimated population density for Senegal in 2010 were obtained from the WorldPop project [40].

Survey and environmental data were linked in ArcGIS 10.3. (ESRI Inc., Redlands CA, USA) based on the WGS-1984 Web Mercator projection at 250m x 250m resolution. A nearest neighbor approach was applied to resample raster data sets Input grids were either extended or clipped to match the geographic extent of a map of the study area, and eventually aligned to it.

Spatial correlation of village seroprevalence levels

Semivariogram analysis of village seroprevalence and village random-effects, adjusting for population density, did not show any spatial dependency in village infection levels (Supplementary Figure 1). The absence of spatial correlation was further confirmed by Bayesian geostatistical modelling using Winbugs software [38]. Including a spatial exponential decay function for village-level random effects did not improve model fit (DIC non-spatial 1333, DIC spatial 1339).

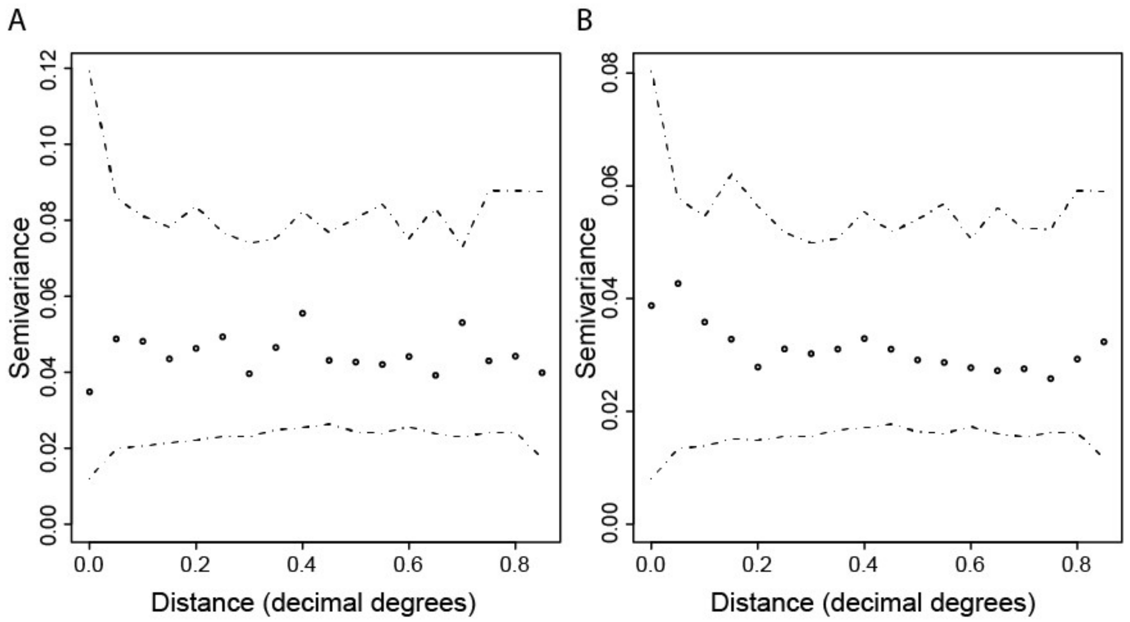

Supplementary Figure 1. (A) Semivariogram of CHIKV village prevalence and (B) village random effects adjusting for log-transformed population density. Envelopes to assess significance of spatial dependency were computed by simulating 1000 permutations.
